# The Impact of Selected Essential Oils Applied to Non-Woven Viscose on Bacteria That Cause Lower Urinary Tract Infections—Preliminary Studies

**DOI:** 10.3390/molecules26226854

**Published:** 2021-11-13

**Authors:** Emilia Frydrysiak, Alina Kunicka-Styczyńska, Krzysztof Śmigielski, Michał Frydrysiak

**Affiliations:** 1 Faculty of Biotechnology and Food Sciences, Institute of Natural Products and Cosmetics, Lodz University of Technology, Stefanowskiego 4/10, 90-924 Lodz, Poland; 2 Faculty of Biotechnology and Food Sciences, Institute of Fermentation Technology and Microbiology, Lodz University of Technology, Wólczańska 171/173, 90-924 Lodz, Poland; alina.kunicka@p.lodz.pl; 3 Department of Environmental Biotechnology, Faculty of Biotechnology and Food Sciences, Lodz University of Technology, Wólczańska 171/173, 90-924 Lodz, Poland; 4 Department of Knitting Technology and Textile Machines, Institute of Fermentation Technology and Microbiology, Lodz University of Technology, Żeromskiego 116, 90-924 Lodz, Poland; michal.frydrysiak@p.lodz.pl

**Keywords:** essential oils, biotextronics system, pantiliner, lower urinary tract inflammations

## Abstract

Inflammation of the lower urinary tract is a very common problem, which occurs particularly in women. A concept of a biotextronics system for preventive and support treatment of lower urinary tract inflammations was presented. The system includes a non-woven viscose insert for essential oils application. The oils were deposited on the non-woven viscose and incubated in the temperature of 37 °C and served a model for their action in the vapor phase as the element of the biotextronics system. The essential oils used in the research were the following: chamomile (*Matricaria chamomilla* L.), sage (*Salvia officinalis* L. and *Salvia lavandulaefolia*), juniper (*Juniperus communis* L.), thyme (*Thymus vulgaris* L.), and mixtures of chamomile oil with oils of each sage species in a 1:1 ratio. The oils were tested against *Escherichia coli*, *Pseudomonas aeruginosa*, *Staphylococcus epidermidis*, *Staphylococcus saprophyticus*, and *Enterococcus faecalis*. The best inhibitory effect in vapor phase was noted for chamomile essential oil at the lowest concentration (0.054 µL/cm^3^). Both mixtures of chamomile and sage acted antagonistically, lowering the antibacterial activity of the individual oils applied solely. Juniper and *Salvia officinalis* essential oils at the concentrations tested increased the growth of at least one of the bacteria tested. *Salvia lavandulaefolia* Vahl. essential oil inhibited all bacteria, only at the concentration 0.214 µL/cm^3^. The thyme oil, at the concentration 0.054 µL/cm^3^, reduced the growth of all bacterial species tested. Chamomile and thyme essential oils were chosen for further research in the biotextronics pantiliner system.

## 1. Introduction

Inflammation of the lower urinary tract is a very common problem which occurs 50 times more often in women than in men, and, according to statistics, afflicts a half of women, at least once in their lives [1,2,3,4]. The main cause of lower urinary tract inflammations, including nosocomial infections, is infections is *Escherichia coli* [5].

*E. coli*, the bacteria residing in a colon, is the cause of approximately 73–95% of infections, including 53–72% of outside-the-hospital and approximately 50% of inside-the-hospital infections [5]. Other species of microorganisms causing lower urinary tract infections and their occurrences are listed in Table 1.

**Table 1:** Microorganisms associated with inflammations of the lower urinary tract [3,6,7,8].

Microorganisms	Occurrence of Infections
*Escherichia coli*	73–95% of infections, 53–72% of ambulatory infections and 18–57% of in-hospital infections
*Staphylococcus epidermidis*	5–10% of all infections
*Staphylococcus saprophyticus*	Up to 2% of ambulatory infections and up to 4% of in-hospital infections
*Pseudomonas* spp.	Up to 4% of ambulatory infections and 1–11% of in-hospital infections
*Enterococcus* spp.	2–12% of ambulatory infections and 7–16% of in-hospital infections
*Staphylococcus aureus*	Mainly in-hospital infections
*Enterobacter* spp.	3% of all infections, mostly in-hospital
*Klebsiella* spp.	3% of all infections, mostly in-hospital, often returns
*Proteus* spp.	3% of all infections, mostly in-hospital
*Serratia* spp.	Mainly in-hospital infections
*Mycobacterium* spp.	Mainly in-hospital infections; may be spread by blood
*Neisseria gonorrhoeae*	Spread by unprotected sex
*Chalmydia trachomatis*	Spread by unprotected sex
*Candida albicans*, *Cryptococcus neoformans*, *Aspergillus* spp.	May be spread by blood

**Figure 1:**
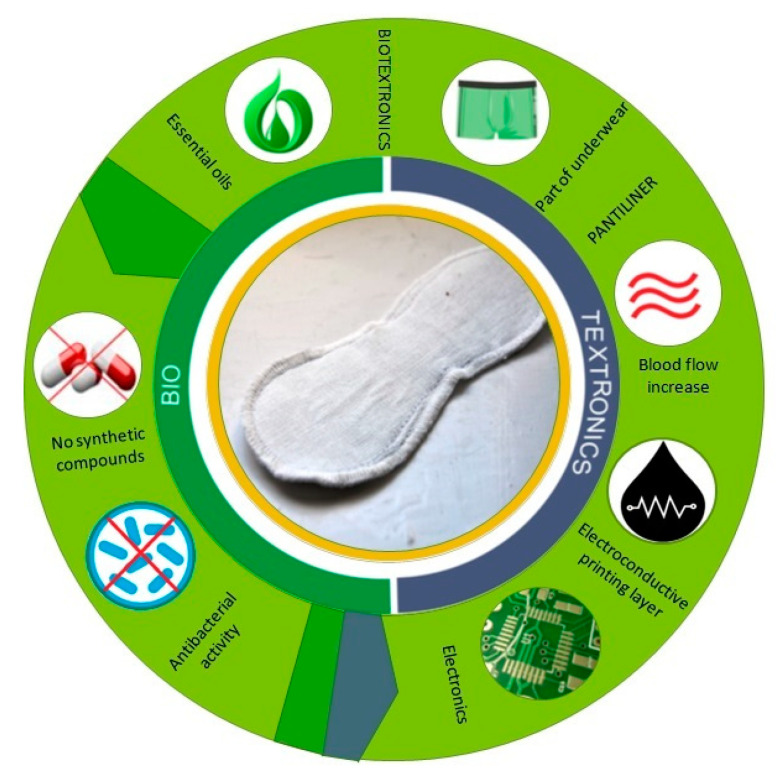
Functionality of the biotextronics system.

The treatment of lower urinary tract inflammations includes not only antibiotics or nitrofurantoin-based medicines, but also hip baths or steam baths with essentials oils, which are recommended by doctors to be taken from several times a week, to 2–3 times a day in the initial phase of the treatment. Hot water or steam increases blood flow, which helps the treatment, and essential oils added to the hot water support healing and relieve discomfort [2,9,10,11,12]. The treatment should be conducted for 15–20 min, which is uncomfortable and difficult to realize day-to-day. That is why the idea of a biotextronics system for protection and support of the lower urinary tract inflammation treatment, facilitating mobility, came to be. The biotextronics system combines essential oils with antibacterial activity, serving as bioactive elements of natural origin and electroconductive printing layers, enabling heating of the pantiliner. Essential oils, with antimicrobial and anti-inflammatory action, in combination with thermal action, alleviate the symptoms of the disease and reduce the multiplication of bacteria (Figure 1).

**Figure 2:**
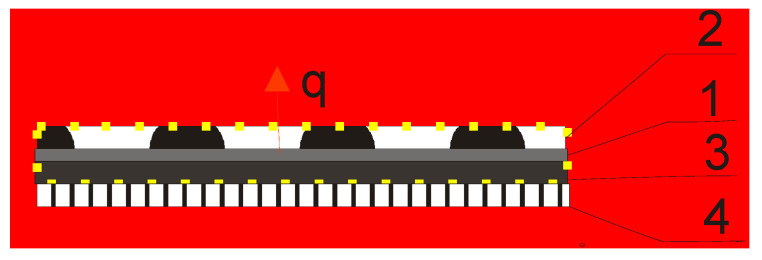
Diagram of the clothing package of the textronics system: 1—textronics heating insert; 2—outer insulating layer, made of non-woven viscose; 3—inner insulating layer made of woolen fabric; 4—a layer of a personal underwear; q—heat flux.

The system consists of a pro-medical underwear equipped with a regulation unit linked to a textronics heating insert and integrated with a biotextronics pantiliner with essential oils by a textile signal line. The biotextronics pantiliner consists of four main parts as follows: two insulating layers and the system heating element placed between them (Figure 2). The pantiliner maintains a constant temperature, thanks to the textronics heating insert. The pantiliner itself is a replaceable element, fixed to this personal pro-medical underwear. The higher temperature makes the essential oil release from the outer part of the biotextronics pantiliner and increases blood flow, which helps the treatment.

The essential oils chosen for the research express antimicrobial activity against a variety of microorganisms including pathogens.

*Salvia officinalis* L. essential oil was proven to have antibacterial and bacteriostatic effects against *E. coli* species concentrations of 1:100 and 1:1000. In the conducted tests, the oil was also active against microorganisms of the following genus: *Aeromonas, Bacillus, Enterococcus, Klebsiella, Listeria, Micrococcus, Proteus, Pseudomonas, Salmonella*, and *Staphylococcus*. Research showed that this essential oil is more active against gram-positive than gram-negative bacteria and its antimicrobial effect is related to the presence of camphor, *α*- and *β*-thujones and 1,8-cineole. The other compounds, *α*- and *β*-pinene, are also characterized by antimicrobial activity [13,14,15,16].

*Salvia lavandulaefolia* Vahl. essential oil is active against the following gram-negative bacteria: *E. coli* (MIC 3.4 mg/mL), *Klebsiella pneumoniae* (MIC 6.9 mg/mL), *Serratia marcescens* (MIC 6.9 mg/mL), *Salmonella cholearesuis* (MIC 4.6 mg/mL), and *Shigella flexneri* (MIC 9.2 mg/mL); and the gram-positive bacteria: *Bacillus subtilis* (MIC 3.4 mg/mL), *Enterococcus faecalis* (MIC 4.6 mg/mL), *Micrococcus luteus* (MIC 2.8 mg/mL), *Staphylococcus aureus* (MIC 2.3 mg/mL), *S. epidermidis* (MIC 2.0 mg/mL), and *Streptococcus mutans* (MIC 2.9 mg/mL) [17]. The antibacterial activity of the oil is mainly attributed to the presence of 1,8-cineole, *β*-thujone, camphor, borneol, and *p*-cymene [18].

*Matricaria chamomilla* L. essential oil is recognized from its strong activity against the following gram-positive bacteria: *S. aureus* (MIC 0.021 µL/mg), *Bacillus cereus* (MIC 0.032 µL/mg), and *B. subtilis* (MIC 0.041 µL/mg). A strong antibacterial effect of the chamomile essential oil was also found against the following gram-negative bacteria: *Shigella shiga* (MIC 0.178 µL/mg), *S. sonnei* (MIC 0.195 µL/mg), and bacteria of the genus *Proteus* (MIC 0.161 µL/mg). The highest value of the minimum oil concentration at which bacterial growth inhibition was found was recorded for *Pseudomonas aeruginosa* (6 µL/mg) [19]. The antibacterial properties are mainly due to *α*-bisabolol; however, chamazulene also has antimicrobial activity against gram-positive bacteria [20]. *α*-bisabolol in the oil also has strong antifungal properties against *Candida albicans, Trichophyton mentagrophytes, T. rubrum*, and *Staphylococcus* [20,21].

*Thymus vulgaris* L. essential oil, due to its antibacterial properties, is extensively used since ancient times. The strongest inhibitory effect was found against *Corynebacterium xerosis* (MIC equal or lower than 0.12 mg/mL). *S. aureus, S. epidermidis, E. faecalis, Acinetobacter baumannii, Klebsiella pneumoniae*, and *Pseudomonas stutzeri* (MIC 0.5 mg/mL) also proved to be sensitive to the thyme essential oil. In relation to *E. coli* and *Serratia marcescens*, the effect of the oil was slightly weaker (MIC equal to 1.0 and 2.0 mg/mL, respectively), while the weakest (MIC equal to or above 4.0 mg/mL) was found against *P. aeruginosa* [22]. The thyme essential oil also shows antibacterial activity, against the following bacteria, among others: *Haemophilus influenzae, Streptococcus pyogenes, Streptococcus agalactiae,* and *Mycobacterium smegmatis*. High sensitivity to this oil was also noted for antibiotic-resistant strains of *A. baumannii* [23]. It also has an inhibitory effect on bacteria of the genus *Salmonella* [24]. Thyme oil also exhibits antiviral activity against the *herpes simplex* virus types 1 and 2, as well as antifungal activity against *Aspergillus niger*, *C. albicans*, and *C. tropicalis* [25].

Studies with *Juniperus communis* L. essential oil have shown that it inhibits the growth of gram-positive bacteria, including the following: *B. cereus*, *B. subtilis, E. faecalis, Listeria monocytogenes, S. aureus,* and *S. epidermidis*; and the following gram-negative bacteria: *E. coli, Klebsiella oxytoca, K. pneumoniae, Proteus mirabilis, Salmonella enteritidis, Shigella sonnei, S. marcescens,* and *Yersenia enterocolitica*. The juniper essential oil also shows activity against fungi of the genus *Candida* [26,27].

The aim of the study was to determine the antibacterial activity of five selected commercial essential oils and two their mixtures against the chosen bacteria, associated with urinary tract infections and inflammations. The selection of those essential oils efficient against chosen bacteria associated with urinary tract infections is crucial for the biotextronics pantiliner’s effectiveness. These preliminary studies are focusing on the oil action in the vapor phase. The oils deposited on the non-woven viscose and incubated at the temperature of 37 °C served the model for their action as the element of the biotextronics system. The system has been patented in Poland (PAT.236378-Textronic system for prophylaxis supporting the treatment and inflammation of the lower urinary tract, https://ewyszukiwarka.pue.uprp.gov.pl/search/pwp-details/P.408106 (accessed on 5 May 2014)), it was awarded with the first prize in “Innovation is a woman” competition in Poland and won three gold medals at the international exhibitions of inventions.

## 2. Results and Discussion

### Antibacterial Activity of Essential Oils Applied on Non-Woven Viscose

**Figure 3:**
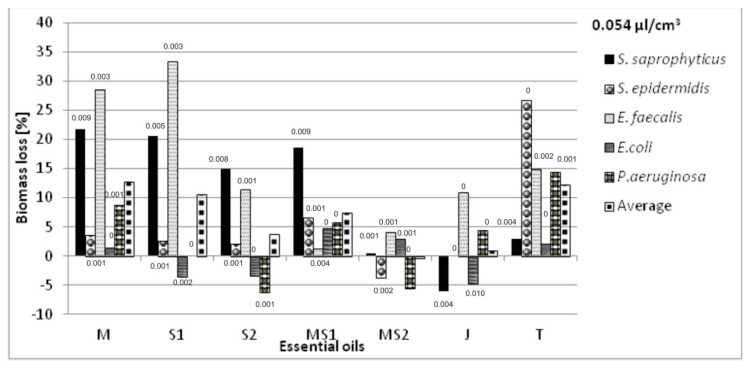
Antimicrobial activity of essential oils in a vapor phase at the oil concentration of 0.054 µL/cm^3^ in the atmosphere, where: M—*Matricaria chamomilla* L.; S1—*Salvia officinalis* L.; S2—*Salvia lavandulaefolia* Vahl.; MS1—*Matricaria chamomilla* L. with *Salvia officinalis* L.; MS2—*Matricaria chamomilla* L. with *Salvia lavandulaefolia* Vahl.; J—*Juniperus communis* L.; T—*Thymus vulgaris* L. Results are presented as an average value of three repetitions with ±SD < 0.01 (values of SD placed above the bars); all the results were statistically different (*p* < 0.05).

**Figure 4:**
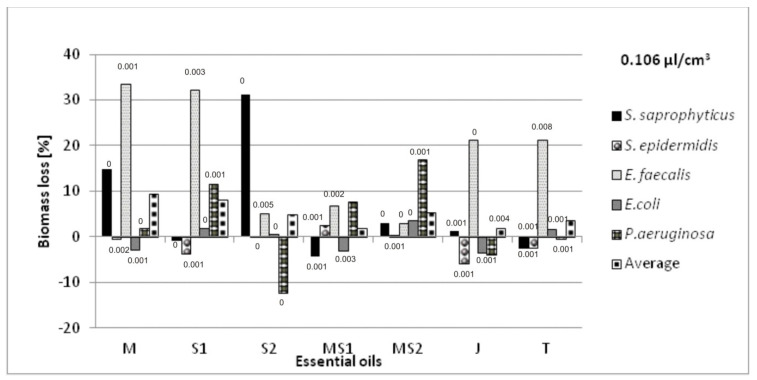
Antimicrobial activity of essential oils in a vapor phase at the oil concentration of 0.106 µL/cm^3^ in the atmosphere, where: M—*Matricaria chamomilla* L.; S1—*Salvia officinalis* L.; S2—*Salvia lavandulaefolia* Vahl.; MS1—*Matricaria chamomilla* L. with *Salvia officinalis* L.; MS2—*Matricaria chamomilla* L. with *Salvia lavandulaefolia* Vahl.; J—*Juniperus communis* L.; T—*Thymus vulgaris* L. Results are presented as an average value of three repetitions and with ±SD < 0.01 (values of SD placed above the bars); all the results were statistically different (*p* < 0.05).

**Figure 5:**
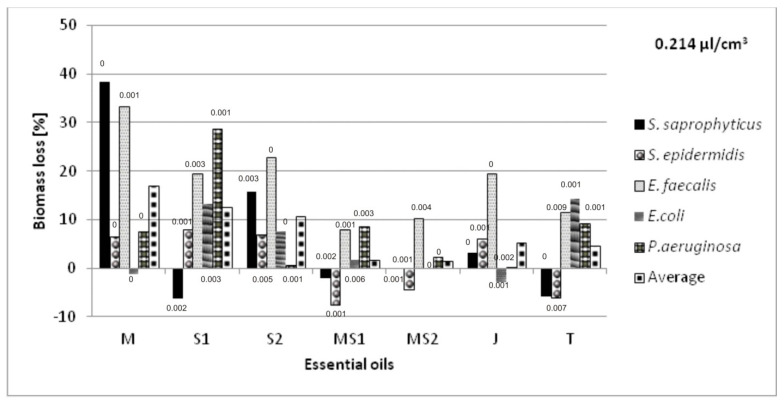
Antimicrobial activity of essential oils in a vapor phase at the oil concentration of 0.214 µL/cm^3^ in the atmosphere, where: M—*Matricaria chamomilla* L., S1—*Salvia officinalis* L., S2—*Salvia lavandulaefolia* Vahl., MS1—*Matricaria chamomilla* L. with *Salvia officinalis* L., MS2—*Matricaria chamomilla* L. with *Salvia lavandulaefolia* Vahl., J—*Juniperus communis* L., T—*Thymus vulgaris* L. Results are presented as an average value of three repetitions with ±SD < 0.01 (values of SD placed above the bars); all the results were statistically different (*p* < 0.05).

The antibacterial volatile activity of five essential oils—chamomile *Matricaria chamomilla* L., sage *Salvia officinalis* L., *Salvia lavandulaefolia* Vahl., juniper *Juniperus communis* L., and thyme *Thymus vulgaris* L.—against five bacteria associated with urinary tract infections were estimated by the microatmosphere method, with three different oil concentrations in the air (0.054; 0.106 and 0.214 µL/cm^3^). The results are presented in Figure 3, Figure 4 and Figure 5. The results were various for each bacterium, relative to the same essential oil. *Salvia officinalis* L. essential oil inhibited *E. faecalis* better than other bacteria tested; however, it also stimulated the growth of *E. coli*. We also noticed that the higher concentration of essential oils in the atmosphere did not mean the higher bacteria biomass decrease.

We aimed to select the essential oil applied on non-woven viscose causing the greatest inhibition of bacterial growth, measured as the biomass lost compared with their growth in the cultures without the oils’ vapors. The average loss of biomass for selected oils was also calculated taking into account all the bacteria species. The highest total biomass loss was a criterion for the selection of the essential oils intended for further research in the biotextronics system. The sets of oils’ combinations, with the increase in biomass of at least one strain, were not considered as useful.

The selected essential oils applied on the material have various impacts on individual species. The greatest biomass loss was observed for *Matricaria chamomilla* L. essential oil against *S. saprophyticus* (38%) and *E. faecalis* (33%) in the highest oil concentration. However, in that concentration, it caused a slight increase in biomass of *E. coli* (1%). There was also a significant increase in biomass in the microatmosphere of 0.054 and 0.106 µL/cm^3^ of *Salvia officinalis* L. against *E. faecalis* (33 and 32%, respectively) and *Salvia lavandulaefolia* Vahl. against *S. saprophyticus* (31%) in the microatmosphere of 0.106 µL/cm^3^ (Figure 3 and Figure 4).

Combining chamomile essential oils with two types of sage did not bring satisfactory results. Although this oil combination increases the anti-inflammatory effect [18], the average loss of bacterial biomass was low (from −0.3 to 7%), and at the selected concentrations, it caused an increase in biomass for some bacteria (Figure 3, Figure 4 and Figure 5).

No increase in biomass for any of the tested bacterial strains was recorded at the following atmospheres: *Matricaria chamomilla* L. essential oil, its mixture with *Salvia officinalis* L. essential oil and *Thymus vulgaris* L. essential oil in the concentration of 0.054 µL/cm^3^; *Matricaria chamomilla* L. essential oil mixed with *Salvia lavandulaefolia* Vahl. essential oil in the concentration of 0.106 µL/cm^3^; *Salvia lavandulaefolia* Vahl. essential oil at the concentration of 0.214 µL/cm^3^. The highest average biomass loss with the simultaneous absence of biomass growth was observed for *Matricaria chamomilla* L. and *Thymus vulgaris* L. essential oils at 13 and 12%, respectively.

According to the literature, chamomile essential oil in a vapor phase was effective against *E. coli, P. aeruginosa*, and *S. aureus*, with MIC values 2.19 µg/cm^3^, 1.02 µg/cm^3^, and 1.06 µg/cm^3^, respectively [28]. In our research, vapors of *Matricaria chamomilla* L. essential oil substantially inhibited the growth of all bacteria investigated at the concentration of 0.054 µL/cm^3^.

The activity of *Salvia officinalis* L. essential oil against *E. coli*, *S. saprophyticus*, and *S. epidermidis* was confirmed in another study [29]. In the presented research, the oil, even at the concentration of 0.054 µL/cm^3^, retarded the growth of both *Staphylococcus* species by 20.1 and 2.6%, respectively, as well as *E. coli* by 1.9% at the concentration of 0.106 µL/cm^3^. Its antibacterial activity is caused by the presence of camphor, thujone, and 1,8-cineole. Camphor was also detected in a vapor phase [29,30].

In the disc diffusion method, MICs for *S. lavandulaefolia* Vahl. essential oils were 3.42 mg/mL against *E. coli*, 4.62 mg/mL against *E. faecalis*, and 2.31 mg/mL against *S. aureus* [31]. We noted for both *Staphylococcus* species and the *E. faecalis* investigated that this essential oil was inhibitory in vapor phase at 0.054 µL/cm^3^ and for *E. coli* at 0.214 µL/cm^3^; however, the bacterial growth was reduced from 2.1 (*S. epidermidis*) to 14.9% (*S. saprophyticus*). Although these two methods cannot be compared directly, the result showed the oil action even in much smaller concentrations.

Due to the lack of literature data on the antimicrobial activity of juniper essential oil in vapors we noted its activity, measured by the disc diffusion method, where *Juniperus communis* L. essential oil inhibited the growth of *S. epidermidis* and *E. faecalis*, with no effect on *E. coli* [27]. In the investigation presented, in the vapor phase at 0.054 µL/cm^3^, the juniper oil reduced the growth of *E. faecalis* by 10.8%, and at 0.214 µL/cm^3^, minimized *E. faecalis* development by 19.6%, but did not inhibit the growth of *E. coli* in any of the investigated concentrations.

The volatile compounds, such as linalol, bornyl acetate, limonene, and γ-terpinolene of thyme essential oil are attributed to its biological activity. According to the literature, the MIC of thyme oil in vapor phase against *S. aureus* was 0.26 µL/cm^3^ [28], while in the presented research, the thyme oil at its lowest concentration 0.054 µL/cm^3^ reduced the growth of two species of *Staphylococcus*—*S. saprophyticus* and *S. epidermidis* by 2.9 and 26.7%, respectively. *Thymus vulgaris* L. essential oil in vapor phase did not bring the expected ceasing of bacterial growth; however, one of its compounds, thymol, is proven to be a highly antiseptic phenol derivative [28].

Essential oils are characterized by a wide spectrum of antimicrobial action. Apart from the tested microbial strains, sage (*Salvia officinalis*) oil is reported to be active against other bacteria associated with lower urinary tract infections, *Enterococcus* sp. and *Klebsiella* sp. [32,33], and expresses antifungal activity against both *Candida albicans* [34] and *Candida* non-albicans pathogenic strains [35]. Thyme oil, known as a strong antibacterial and antifungal agent, is also effective against *Klebsiella pneumoniae* [36] and *C. albicans* [37]. Owing to their antimicrobial activity, the sage and thyme essential oil vapors may be helpful in cases of mixed bacterial–fungal infection of the urinary tract.

Essential oils applied topically may cause skin inflammations and allergic reactions. The research on oils toxicity towards normal human cells are scarce and researchers are rather focusing on their toxicity against cancer cells; however, essential oils may also be considered as skin penetration enhancers for transdermal delivery of drugs [38]. Safe application of oils and their vapors on human tissues relies upon the proper balance between their concentration and the exposition time. Essential oils constituents are proved to penetrate into the bloodstream but are subjected to fast metabolism or excreted with urine and feces without accumulation [38,39,40]. Chamomile essential oil is historically proved as a safe agent in a variety of applications, including topical ones. Aqueous and methanolic extracts of chamomile, tested against human normal cells, did not present adverse effects in contrast to their action against various human cancer cells [41]. Applied against human cancer cell lines, chamomile essential oil was characterized as a weak cytotoxic agent [42] and sage and thyme oils expressed dose-dependent cytotoxicity [43]. Among the oils tested in the present study, thyme essential oil may raise concerns, but it also has long been used in the treatment of respiratory tract infections and in the relief of pruritus associated with dermatitis and bruises. High concentrations of thyme oil may be a cause of skin irritation; however, no toxicity has been reported after its administration in lower doses [44].

## 3. Materials and Methods

### 3.1. Material for Essentials Oils Application

The part of the biotextronics pantiliner used in the research was a non-woven viscose material. Its characteristics are presented in Table 2. It originated from Lentex S.A. (Lubliniec, Poland).

### 3.2. Essential Oils

In the study, the five following commercial essential oils were used: *Matricaria chamomilla* L., *Salvia officinalis* L., *Salvia lavandulaefolia* Vahl., *Juniperus communis* L., and *Thymus vulgaris* L.; in addition, mixtures of *Matricaria chamomilla* L. with each sage species in a 1:1 ratio (*v*/*v*) were also used. Essential oils were purchased from Avicenna-Oil^®^ (Wrocław, Poland) and fulfilled the requirements of good manufacturing practices. Characteristics of the essential oils are shown in Table 3.

### 3.3. Microorganisms and Antibacterial Activity Assessment of Essential Oils

Five bacterial strains were used as follows: *Enterococcus faecalis* (isolated from water and identified in the Institute of Fermentation Technology and Microbiology, Lodz University of Technology, Żeromskiego, Poland), *Escherichia coli* ATCC10536, *Pseudomonas aeruginosa* ATCC15442, *Staphylococcus epidermidis* ATCC12228, and *Staphylococcus saprophyticus* DSM4853. The strains designated ATCCs originated from American Type Culture Collection and a DSM strain was obtained from Leibniz Institute DSMZ—Deutsche Sammlung von Mikroorganismen und Zellkulturen, GmbH. The microorganisms were activated through double passaging on TSA medium (trypticase soy agar) Oxoid, UK (37 °C, 48 h).

#### Microatmosphere Method

The essential oils’ activity in the vapor phase was determined by the microatmosphere method. The suspensions of the tested bacteria were prepared in a physiological salt solution (NaCl 8.5 g/L), later standardized to the density of about 10^8^ CFU/mL, and in the amount of 0.03 mL of a particular microorganism, these were transferred onto the TSA medium. Non-woven viscose discs of 55 mm in diameter were soaked with 0.5 mL of an appropriate essential oil in DMSO solution, which resulted in three different microatmospheres (0.054; 0.106 and 0.214 µL/cm^3^). The discs were placed on the lid of the plate (inhibition in the gas phase) and cultured upside-down. During culturing, the discs were on the bottom lid. Subsequently, the plates were secured with Petri film. Petri dishes were kept at 4 °C for 2 h, and then incubated at 37 **°**C for 24 h. After incubation, each agar medium with inoculated bacteria was transferred into 50 mL of sterile water to rinse the biomass off, and the optical density (OD) of the suspension was measured at the wavelength 560 nm with the use of TriStar2S LB942 spectrophotometer (Berthold Technologies, Bad Wildbad, Germany). The reference sample was the specific bacteria culture grown without the essential oil. Positive controls were the bacteria cultures with the non-woven viscose discs soaked with 0.5 mL of 96% ethanol solution. Antimicrobial activity of DMSO was also checked analogically and no adverse effect on the tested microorganisms was observed. The experiments were conducted in triplicate and results were presented as an average value with a standard deviation.

The essential oils vapor activity against bacteria was expressed as a percent of biomass loss according to Formula (1), as follows:

(1) $$I\left(\%\right)=\frac{A_{s}-A_{b}}{A_{s}}\cdot 100\%$$

where *I*—biomass loss (%); *A_s_*—an absorbance at 560 nm of the biomass suspension of the bacteria grown without the essential oil; *A_b_*—an absorbance at 560 nm of the biomass suspension of the bacteria grown in the microatmosphere of the essential oil vapors.

### 3.4. Statistical Analysis

All of the results were expressed as the mean ± SD of three independent experiments. One-way ANOVA was used in order to compare the statistical differences (*p* < 0.05).

## 4. Conclusions

The best inhibitory effect in vapor phase was noted for *Matricaria chamomilla* L. essential oil at the lowest concentration (0.054 µL/cm^3^). Both mixtures of chamomile (*Matricaria chamomilla* L.) essential oil with two species of sage (*Salvia officinalis* L. and *Salvia lavandulaefolia* Vahl.) acted antagonistically, lowering the antibacterial activity expressed by the individual oil applied solely. Juniper and *Salvia officinalis* essentials oils, at the concentrations tested, increased the growth of at least one of bacteria species. *Salvia lavandulaefolia* Vahl. essential oil inhibited all bacteria only at the highest concentration 0.214 µL/cm^3^. This essential oil is also socially accepted and, in low concentrations, is used to alleviate irritations to human skin. The thyme oil (*Thymus vulgaris* L.), at its lowest concentration 0.054 µL/cm^3^, reduced the growth of all bacterial species. However, both chamomile and thyme essential oils were chosen for further research in the biotextronics pantiliner system.

## Figures and Tables

**Table 2:** Characteristic of the non-woven viscose insert.

Material	Function	Surface Mass [g/m^2^]	Thickness [mm]
Non-woven viscose	External, removable insert	30	0.2

**Table 3:** Characteristics of essential oils.

*Matricaria chamomilla* L. Essential Oil	
Organoleptic Description	clear, viscous liquid, dark blue, with a characteristic odor
Analytical Data	density (at 20 °C): 0.946 to 0.969 g/cm^3^ refractive index (at 20 °C): 1.496–1.516
Chromatographic Profile	(−)-α-bisabolol: 10–65% chamazulene: ≥1.0% bisabolol oxide and (−)-α-bisabolol: ≥20%
***Salvia officinalis* L. Essential Oil**	
Organoleptic Description	clear liquid, slightly yellow or slightly green, with a characteristic odor
Analytical Data	density (at 20 °C): 0.905 to 0.925 g/cm^3^ refractive index (at 20 °C): 1.457 to 1.473 optical rotation (at 20 °C): −3.0° to +15.0°
Chromatographic Profile	1,8-cineole: 6.0–16.0% α- and β-thujone: 20–40% camphor: 14.0–37.0% bornyl acetate: max. 5.0% borneol: max 5.0%
***Salvia lavandulaefolia* Vahl. Essential Oil**	
Organoleptic Description	clear liquid, light yellow, with a characteristic odor
Analytical Data	density (at 20 °C): 0.907 to 0.927 g/cm^3^ refractive index (at 20 °C): 1.458 to 1.478 optical rotation (at 20 °C): +9° to +19° flash point: 54 °C
Chromatographic Profile	α-thujone: 30.3% camphor: 20.3% 1,8-cineole: 12.5% α- and β-pinene: 0.6–5.9% borneol: 2.7% α- and β-caryophyllene: 1.6–2.7% bornyl acetate 1.9% α-terpineol: 1.2%
***Juniperus communis* L. Essential Oil**	
Organoleptic description	Clear liquid, colorless or slightly yellow, with a characteristic odor
Analytical Data	density (at 20 °C): 0.857 to 0.876 g/cm^3^ refractive index (at 20 °C): 1.471–1.483 optical rotation (at 20 °C): −15° to −0.5°
Chromatographic Profile	α-pinene: 20.0–50.0% sabinen: ≤20.0% β-pinene: 1.0–12.0% β-myrcene: 1.0–35.0% α- phellandrene: ≤1.0% limonene: 2.0–12.0% terpinen-4-ol: 0.5–10.0% bornyl acetate: ≤2.0% β-caryophyllene: ≤ 7.0%
***Thymus vulgaris* L. Essential Oil**	
Organoleptic Description	clear, yellow to dark red-brown liquid, with a strong odor of thymol
Analytical Data	density (at 20 °C): 0.915 to 0.935 g/cm^3^ refractive index (at 20 °C): 1.490 to 1.505 optical rotation: −7° to +3° flash point: 58 °C
Chromatographic Profile	α-thujen: 0.2–1.5% β-myrcene: 1.0–3.0% α-terpinene: 0.9–2.6% ρ-cymene: 14.0–28.0% γ-terpinene: 4.0–12.0% linalool: 1.5–6.5% terpinen-4-ol: 0.1–2.5% methyl carvacrol ether: 0.05–1.5% thymol: 37.0–55.0% carvacrol: 0.5–5.5%

## Sample Availability

Samples of the essential oils are available from the authors.
